# Ex Vivo Expansion of Hematopoietic Stem and Progenitor Cells from Umbilical Cord Blood

**Published:** 2016

**Authors:** E.V. Sotnezova, E.R. Andreeva, A.I. Grigoriev, L.B. Buravkova

**Affiliations:** Institute of Biomedical Problems of the Russian Academy of Sciences, Khoroshovskoye shosse 76A, Moscow, 123007, Russia

**Keywords:** cord blood, hematopoietic stem cells, ex vivo expansion

## Abstract

Transplantation of umbilical cord blood cells is currently widely used in modern cell therapy. However, the limited number of hematopoietic stem and progenitor cells (HSPCs) and prolonged time of recovery after the transplantation are significant limitations in the use of cord blood. *Ex vivo *expansion with various cytokine combinations is one of the most common approaches for increasing the number of HSPCs from one cord blood unit. In addition, there are protocols that enable *ex vivo *amplification of cord blood cells based on native hematopoietic microenvironmental cues, including stromal components and the tissue-relevant oxygen level. The newest techniques for *ex vivo *expansion of HSPCs are based on data from the elucidation of the molecular mechanisms governing the hematopoietic niche function. Application of these methods has provided an improvement of several important clinical outcomes. Alternative methods of cord blood transplantation enhancement based on optimization of HPSC homing and engraftment in patient tissues have also been successful. The goal of the present review is to analyze recent methodological approaches to cord blood HSPC *ex vivo *amplification.

## INTRODUCTION

At the end of the last century, umbilical cord blood (UCB) attracted the interest of researchers and physicians in the field of bone marrow transplantation due to its successful use as an alternative source of hematopoietic cells. Currently, UCB is used for more than just hematological transplantations. The list of diseases and pathologies which can be treated with UCB is expanding every year. It should be noted that UCB contains blood cells of different commitment, including mature blood elements and hematopoietic stem and progenitor cells (HSPCs), as well as other cell types: undifferentiated somatic stem cells [1-5], multipotent mesenchymal stromal cells (MSCs) [6-9], and endothelial progenitor cells [10].

As a hematopoietic tissue transplant, cord blood has the following undisputable advantages: a non-invasive method of collection, availability, and safety for a donor and lower incidence and severity of “graft-versushost” reactions compared to the bone marrow or mobilized peripheral blood [11-13]. However, due to a low content of HSPCs, UCB also has some disadvantages associated with the slow recovery of hematopoiesis and immunity. UCB substantially differs from that of bone marrow or mobilized peripheral blood in quantity, composition, and properties of hematopoietic cells. In contrast to bone marrow HSPCs, UCB HSPCs are outside of the cell cycle, but they have a pronounced and rather fast proliferative response to growth factors stimulation [14-17]. The ability of UCB HSPCs to expand *ex vivo *in response to stimulation became the basis for the development of different approaches towards increase of the HSPC number in UCB transplants.

There are two main strategies to enrich the HSPC number in a UCB mononuclear fraction: the first one is based on the expansion of committed hematopoietic progenitors and the other one, on increasing the number of cells with a high proliferative potential, HSPCs [18]. In the first case, the use of committed cells reduces the duration of hematopoietic recovery after transplantation, while the second one eliminates the need for an additional unit of UCB. For example, successful long-term recovery of hematopoiesis after bone marrow aplasia with *ex vivo *expanded committed progenitors requires the administration of an additional unit of UCB which has not been subjected to any manipulations and contains HSPCs. However, if the *ex vivo *expansion provides cells that are capable of long-term support of the hematopoiesis (long-term repopulating cells), then further manipulations will produce both undifferentiated and committed cells, which can guarantee short-term and long-term recovery of hematopoiesis after the transplantation. This approach does not require the administration of an additional unit of UCB. It is worth noting that in addition to the approaches described above, there are other strategies to improve the efficiency of UCB application that are not aimed at expansion, but focus on enabling effective homing and engraftment of the transplanted cells [19-25].

## BASIC APPROACHES TO EX VIVO EXPANSION OF UMBILICAL CORD BLOOD HSPCs

**Fig. 1:**
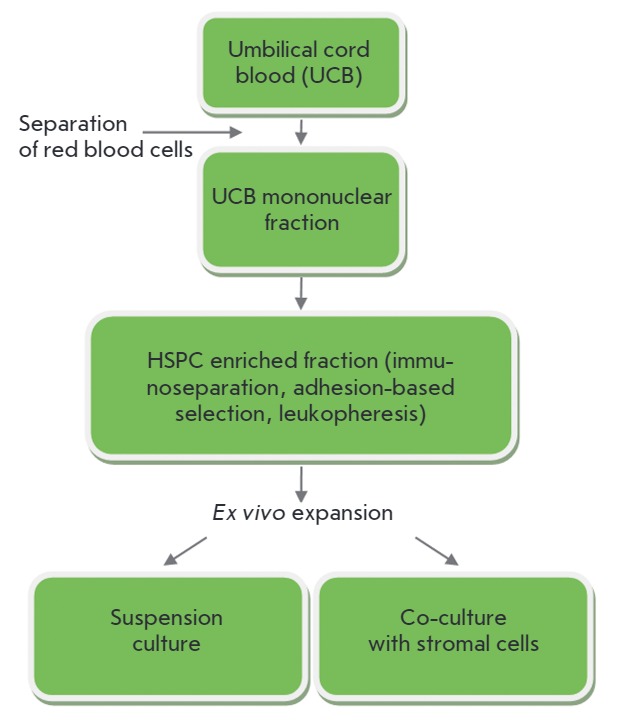
Methodological approaches for *ex vivo *expansion of cord blood cells

The development of effective and controlled approaches to generating a large number of HSPCs focuses primarily on the selection of growth media components and methods for the isolation of undifferentiated cells. However, most of the existing models for culturing HSPCs from UCB underestimate the importance of the local microenvironment: interactions with stromal elements, paracrine regulation, and oxygen concentration (*Fig. 1*).

**Use of enriched fractions of UCB**

The choosing of an approach to the expansion of umbilical cord blood cells starts with the choice between the use of an unfractionated hematopoietic tissue sample and conducting a selection. The separation of HSPCs is performed using magnetic or fluorescent-labeled monoclonal antibodies against specific antigens. It is possible to use either a positive (isolation of certain types of cells from heterogeneous initial material) or negative (unwanted cells are removed from the suspension) selection. It has been shown that the use of a fraction enriched in hematopoietic cells leads to better outcomes of expansion in vitro [26].

CD34 and CD133 are the most common markers for a positive selection of hematopoietic stem cells, but their use excludes from the expansion cells that are negative for these antigens, but possess stem cells properties [27]. The presence of certain surface markers is not indicative of the physiological features of a cell, such as its capacity for self-renewal, proliferation, or differentiation. In addition, the expression of a phenotype may be unstable. For example, Summers et al. have shown that a population of CD34^-^Lin^-^ umbilical cord blood cells generates CD34^+^-HSPCs in co-culture with murine bone marrow stromal cells [28]. This approach has other disadvantages, as well: it requires a large number of initial cells and some hematopoietic cells are lost during the isolation [29]. A decision to forgo prior immune separation prevents potential cellular damage during numerous laboratory manipulations (centrifugation, resuspension, etc) and changes in the functional state of the cells provoked by the binding of antibodies to surface molecules [30].

Some studies have applied unfractionated UCB in HSPC expansion [31-33]. There are also approaches in which one portion of a cord blood unit is administered to a recipient without any treatment, while the other portion is used for expansion with prior enrichment (CD34^+^ or CD133^+^ selection). In this approach, the graft retains its immunological potential, which improves its engraftment and immunological restoration [34, 35].

**Soluble components of culturing systems**

Fetal calf serum (FCS), which contains a natural cocktail of growth factors, adhesion mediators, minerals, lipids and hormones, is the standard component for cultivation of most human cell types, including HSPCs. However, there is no consensus on the possibility of using cells after FCS-supported expansion in clinical settings. The disadvantages of a serum include difficulties in standardization of its composition, potential viral contamination, and high risk of immunization of a recipient with foreign proteins [36, 37]. Therefore, some researchers reject FCS in favor of cytokine cocktails [26, 38]. Nevertheless, it should be taken into account that the serum contains some minor components whose exact activity has not yet been identified and, therefore, may not be fully compensated for in serum-free media.

Numerous soluble factors that affect the proliferation and differentiation of HSPCs have been identified to date. Their various combinations define the timing and degree of expansion of the cultured cells. Both peripheral blood cells and UCB cells synthesize cytokines. In particular, UCB T-cells, NK-cells, and macrophages produce a granulocyte colony-stimulating factor (GCSF), a granulocyte-macrophage colony-stimulating factor (GM-CSF), a macrophage colony-stimulating factor, interleukins 2, 3 and 4 (IL-2 -3, -4), transforming the growth factor (TGF-β), and interferon-γ [39-41]. However, the amount of the mediator synthesized, its biological activity, and the number of producing cells are considerably lower in UCB than in peripheral blood.

Despite the abundance of recombinant cytokines that are used for the expansion of primitive hematopoietic progenitors, no optimal combination has yet been approved for use in clinical practice. The most commonly used factors are the stem cell factor (SCF), IL-3 and -6, G-CSF, thrombopoietin (TPO), and Flt-3 ligand [42, 43].

It should be noted that in addition to the set of factors, their concentration and the sequence of their use are also important. For example, cultivation of HSPCs during the first three days using SCF, IL-3, Flt-3, TPO, in 4% fetal calf serum, followed by transfer into a medium with 20% fetal calf serum and macrophage colony stimulating factor, Flt-3, IL-3, and SCF promotes the expansion of CD34^+^ cells [43]. Growth factors SCF, Flt- 3, IL-11, IL-3, IL-6, GM-CSF are responsible for cell proliferation, whereas the macrophage-colony-stimulating factor, G-CSF, erythropoietin (EPO), and TPO are responsible for cell differentiation and maturation. SCF, IL-3, and IL-6 act in the G0/G1 phase of the cell cycle and collectively induce mitosis [44].

Other combinations of cytokines are also used for the expansion of hematopoietic cells. Haylock et al. showed that expansion with a combination of IL-lβ, IL-3, IL-6, G-CSF, GM-CSF, and SCF is more effective than without one of these six cytokines [45].

It should be noted that there are factors whose presence in the culture medium reduces the expansion of hematopoietic cells. It has been shown that IL-8, platelet factor-4, protein induced by IFN-γ, and monocyte chemotactic factor-1 downregulate *in vitro *proliferation of colony-forming units of granulocytes, erythrocytes, monocytes and megakaryocytes (CFU-GEMM), granulocytes and monocytes (CFU-GM), and burstforming units of red blood cells (BFU-RBC), stimulated by growth factors [46, 47]. Also, macrophage inflammatory protein-α inhibits the proliferation of murine stem cells, corresponding to CFU-S 12 (colony forming units of the spleen, which give rise to granulocytic, monocytic, erythroid, megakaryocytic and lymphoid colonies on Day 12 after the transplantation into irradiated animals) and earlier CFU-Bl cells (cells forming blast cell colonies in the culture) in mice in an *ex vivo *system [48].

Culture systems that contain only soluble factors deprive hematopoietic cells of the supporting influence of the microenvironment: cell interactions with nonhematopoietic cells, components of the tissue matrix, and paracrine mediators. On the other hand, the addition of exogenous cytokines into stroma-based cocultures where feeder cells produce SCF and IL-6, as well as many other paracrine factors, may promote the maintenance of hematopoietic progenitors, but this is not strictly mandatory.

## MODELING A SPECIFIC MICROENVIRONMENT FOR EX VIVO EXPANSION OF UCB HSPCS

It should be mentioned that early studies of adult hematopoietic stem cells have been associated with modeling of their natural microenvironment [49, 50]. For example, the initial attempts to cultivate hematopoietic cells in suspension cultures demonstrated a rapid decline of hematopoiesis and replacement of hematopoietic cells with macrophages. The use of a culturing system comprising the bone marrow cell layer, however, yielded a culture containing hematopoietic progenitors possessing the properties of intact bone marrow hematopoietic stem cells [49]. Further studies were focused on the development of various modifications and improvement of the cultivation system.

**Co-cultivation with stromal cells**

Co-culturing with stromal feeder cells is a more physiological alternative to the application of recombinant cytokines, which had been used since the beginning of bone marrow hematopoietic cell studies [49]. Researchers are actively looking for new cell lines that support* in vitro *expansion of HSPCs during co-cultivation [51]. Co-culturing of hematopoietic progenitors with different types of cells which exhibit feeder properties towards them is not only useful for the expansion of undifferentiated precursors for their subsequent clinical use, but also allows one to elucidate the relationship between the cells within the hematopoietic niche.

The traditional and most rational approach to the expansion of HSPC *in vitro *is to use mesenchymal stromal cells as a feeder layer [52-59]. Besides the feeder properties, MSCs have high proliferative activity and are more accessible than other types of human feeder cells (such as ductal epithelial cells or splenocytes) [60]. It has been shown that in Dexter-cultures bone marrow stromal cells can support hematopoiesis *in vitro *for more than 6 months [49]. Some researchers use MSCs after differentiation into osteoblasts, thus creating a semblance of an endosteal niche [61].

MSCs and more differentiated stromal cells secrete various cytokines [62-64]. Almost all data on cytokine production by human MSCs are collected *in vitro*; therefore, it is impossible to state with any degree of confidence how each cytokine is involved in paracrine regulation *in vivo*. Nevertheless, it has been shown that MSCs produce large amounts of cytokines that support resting or self-renewed HSPCs, in particular SCF, a leukemia cell inhibitory factor, stromal cell-derived factor 1 (SDF-1), oncostatin M, morphogenetic bone protein-4 , Flt-3 ligand, and TGF-β, IL-1, -6, -7, -8, -11, -12, -14, -15 [62, 63]. Furthermore, when IL-1α is added to the culture medium, MSCs can produce growth factors, such as GM-CSF and G-CSF, which affect more mature hematopoietic precursors, indicating mutual regulation of hematopoietic cells and MSCs [65-67].

Stromal precursors from different sources are applied in in vitro modeling of bone marrow niche conditions [52, 54, 68]. MSCs from the bone marrow are the most commonly used, and, therefore, they are well characterized as feeder cells. MSCs have also been derived from the walls of blood vessels, the synovial membrane, placenta, umbilical cord blood, and the subendothelial layer of the umbilical vein. MSCs from different sources differ in the expression of some markers, their ability to proliferate and differentiate, but in general their characteristics are similar [69-72]. MSCs from the stromal-vascular fraction of human adipose tissue have been shown to support hematopoiesis in vitro [53, 73]. Therefore, they are a good alternative to bone marrow MSCs and represent an easily accessible source of feeder cells for the expansion of UCB HSPCs for widespread clinical use [74].

McNiece *et al*. developed a protocol for culturing HSPCs according to which 14-day expansion includes 7 days of co-cultivation with bone marrow MSCs in the presence of hematopoietic cytokines, followed by 7 days of culturing in the presence of cytokines alone [52]. This technique significantly reduces the neutrophils and platelets recovery time after transplantation of two units of UCB, one of which is enriched with HSPCs using the protocol above. Thus, the use of feeder layers for expansion of UCB HSPCs allows one to exclude exogenous growth factors that reduce the efficiency of cell amplification.

**Tissue-related oxygen level**

Oxygen concentration is one of the main factors of hematopoietic microenvironment that is involved in the regulation of hematopoietic cell development. The oxygen level in bone marrow varies from 1 to 6%; the hypoxic areas contain resting HSPCs, whereas proliferating HSPCs are located in sites with higher O_2_ [75]. Low partial oxygen pressure plays an important role in the maintenance of certain physiological properties of hematopoietic cells, which is important for studies of stromal and hematopoietic cells interactions *in vitro*, and must always be taken into consideration when designing amplification protocols for UCB cells [76, 77].

A lower oxygen level is known to have a significant effect on hematopoietic cells *in vitro*, affecting their colony-forming ability, resistance to radiation, and their potential to restore hematopoiesis in lethally irradiated animals [75, 78]. Additionally, low partial oxygen pressure promotes the viability and proliferation of undifferentiated hematopoietic cells over committed progenitors [78, 79].

Remarkably, a combination of different O_2_ concentrations and cytokine sets results in amplification of UCB cells with different properties. For example, Ivanovic* et al*. have shown that the application of 3% oxygen in the presence of SCF, G-CSF, TPO, and IL-3 supports primitive hematopoietic cells capable of restoring hematopoiesis in irradiated animals after transplantation and contributes to the expansion of committed precursors (CFU) [80].

It has also been shown that cultivation of a UCB fraction enriched with CD133^+^ cells supplemented with the recombinant cytokines SCF, Flt-3, TPO, IL-6, and IL-3 under 5% O_2_ results in an almost 27-fold increase in the number of CD34^+^CD38^-^ cells (irrespective of the presence of serum in the medium), which is significantly (P < 0.01) higher than in the case of a standard oxygen concentration [81]. Cells amplified in low oxygen condition contained more CFU with a myeloid potential and had a higher ability to restore hematopoiesis after transplantation into irradiated animals. It has been shown that a low oxygen level induces the expression of the *HIF-1α*, *VEGF, *and *ABCG2 *genes in hematopoietic cells and activates the expression of CXC chemokine receptor 4 (CXCR4) [82].

Tursky *et al*. cultivated UCB cells at 10% oxygen in a medium supplemented with cytokines (TPO, SCF, Flt-3 ligand and IL-6) and obtained a higher HSPC expansion compared with the most common UCB cell culturing protocol (20% oxygen, TPO, SCF, and G- CSF) [42].

**Fig. 2:**
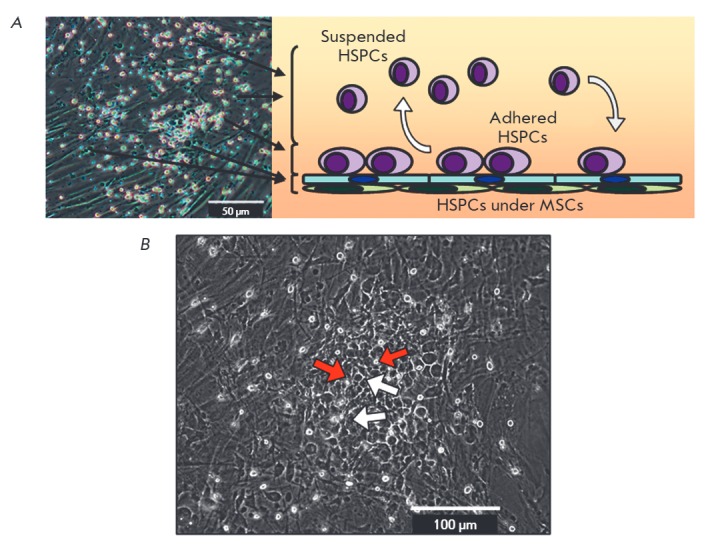
Cord blood mononuclear cultivation on a MSC feeder layer. A. Representative image of HSPC/MSC co-culture and schematic distribution of HSPCs. B. MSC-associated HSPCs in HSPC/MSC coculture. HSPCs attached to the MSC surface (white arrows), “cobblestone” area forming cells beneath the MSCs (red arrows).

One of the important features of hematopoietic cells in occuping certain “niches” when co-cultured with stromal cells is also dependent on the oxygen level. Already in 1977, Dexter *et al*. had described the compartmentalization of hematopoietic cells in such cocultures: some hematopoietic progenitors were present in the suspension above the feeder layer, some adhered to the stromal surface, and some cells migrated to the substromal space (*Fig. 2A*) [49]. Long-term co-cultivation was accompanied by the formation of sites of HSPC active proliferation and the formation of so-called “cobblestones” areas, which are detected by phase-contrast microscopy and look like dense cell clusters under the MSC layer (*Fig. 2B*) [82].

**Fig. 3:**
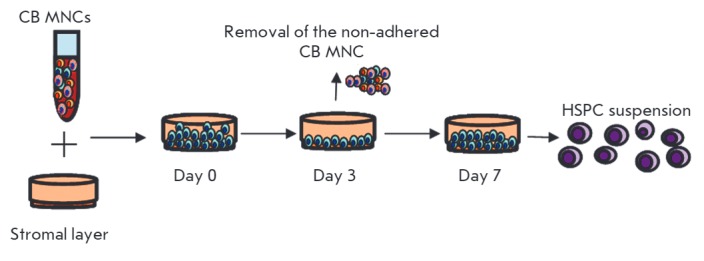
The experimental design of cord blood mononuclear (CB MNC) expansion on a MSC layer, where the adhered fraction of CB MNCs is able to generate a new cell population enriched with HSPCs (Maslova *et al*., 2013).

The spatial organization of hematopoietic cells in a co-culture is comparable to their distribution in bone marrow: based on a state of resting or active proliferation, cells are located in areas with different oxygen levels and nutrients availability. It was assumed that the fraction of cells which adhered to the surface of MSCs was enriched in actively proliferating cells. Compared to other fractions of hematopoietic progenitors in the co-culture, the cells that migrated under the stromal monolayer rarely divided and retained an immature CD34^+^CD38^-^ phenotype [83]. Therefore, the peculiarities of HSPCs distribution in certain compartments based on their proliferative potential can be used for the fractionation of cells according to their ability to adhere and the isolation of populations of cells with certain properties (*Fig. 3*) [73].

According Jing *et al*., the most “hypoxic” hematopoietic cells were localized under the stromal monolayer when cultured under standard (atmospheric) 20% O_2_ [83]. The adhesion of HSPCs to the stromal layer decreased under reduced O_2_, but these conditions promoted the migration of the cells under the MSC monolayer. Hypoxia conditions amplified the production of vascular endothelial growth factor A, which apparently enhanced the permeability of the MSCs monolayer. It should be noted that a reduced oxygen concentration affects both hematopoietic and stromal cells and their interaction [83].

Reconstruction of the bone marrow microenvironment* ex vivo *involves generating a tissue-related oxygen level and the application of feeder layers, in particular MSCs, as a cellular component of the microenvironment [76, 77]. However, it should be taken into account that the reduced O_2_ in the culture medium affects not only the hematopoietic cells, but also MSCs.* In vitro *studies revealed a decrease in the ostegenic and adipogenic differentiating potential of MSCs under hypoxic conditions [84, 85]. Furthermore, a reduced oxygen concentration during cultivation promoted chondrogenic differentiation and an increase in the proliferative activity and the number of fibroblast-colony- forming units [84, 86, 87]. These findings highlight the role of oxygen as an important factor that defines the fate of stromal and hematopoietic cells. It is important to consider the effect of oxygen on the production of biologically active MSC mediators, when these cells are used as a feeder layer for the cultivation of hematopoietic cells. It has been shown that MSC production of such mediators as IL-1β, IL-10, the hepatocyte growth factor, vascular endothelial growth factor, basic fibroblast growth factor, TGF-β, and GM-CSF increases under 4–5% O_2_, while that of the tumor necrosis factor α decreases [64, 88].

Koller *et al*. conducted *in vitro *expansion of hematopoietic cord blood cells using an approach based on the effect of a combination of hematopoietic microenvironment factors [89]. The UCB cells were cultured in the presence or absence of recombinant cytokines and an MSC underlayer at 5 or 20% O_2_. It was found that the use of IL-3/IL-6 allows one to achieve a more efficient expansion of hematopoietic progenitors than IL-1/IL-3 for more than 8 weeks. This effect was enhanced under reduced O_2_. The presence of irradiated stromal cells had no significant effect on the expansion of hematopoietic cells in the presence of cytokines, especially at low oxygen.

The effect on hematopoietic cells may vary depending on the oxygen concentration in the medium. Coculturing of umbilical cord blood mononuclears and bone marrow MSCs at 2% O_2_ promotes a substantially lower production of CD34^+^ cells (25-fold increase vs. 60-, 64- and 92-fold increase at 5, 21, 10% O_2_, respectively, on Day 10). Studies of growth dynamics revealed a higher proliferative rate of the UCB cells cultured at 5, 10, and 21% oxygen than that of those cultured at 2% O_2_ [90].

Therefore, to develop effective and controlled approaches for obtaining large quantities of hematopoietic stem and progenitor cells for transplantation, it is necessary to take into account the particular features of the microenvironment of hematopoietic niches, including the tissue-related oxygen level.

## MOLECULAR GENETIC APPROACHES TO EXPANSION OF HEMATOPOIETIC STEM AND PROGENITOR CELLS FROM UMBILICAL BLOOD EX VIVO

The routine approaches to the expansion of UCB cells are based on data from studies of the effect of cellular and non-cellular hematopoietic microenvironment factors on the HSPC, including the tissue-releated oxygen level, interaction with stromal cells, and paracrine mediators. However, the development of molecular genetic techniques has greatly enhanced our understanding of the mechanisms that mediate the function of hematopoietic niches, thereby allowing us to develop new technological approaches to the amplification of UCB HSPCs.

**Notch-mediated expansion**

A family of Notch ligands and receptors is involved in numerous processes [91-93]. The Notch 1 receptor is found on CD34^+^ hematopoietic progenitors [94]. Moreover, activation of Notch signaling contributes to maintenance of the phenotype of the most primitive hematopoietic stem cells in vitro. This results in a serum-free system for culturing CD34^+^ hematopoietic cells that consist of immobilized Delta1 Notch-ligand and early hematopoietic stem cells cytokines (SCF, TPO, Flt-3 ligand, IL-3 and IL-6) [95]. Ambiguous results were obtained for the transplantation of two units of UCB, one of which was enriched in HSPCs using the Notch-system, during a clinical trial. The use of Notchgraft reduced the neutrophils recovery time; however, after 3 months the hematopoiesis in the recipients was maintained by the other UCB transplant. The observed effect can be explained by two reasons: the loss of cells providing long-term hematopoietic recovery (long-term repopulating cells) during the cultivation and the immune response of the T-cells of the intact UCB transplant [34, 35].

**Expansion in the presence of StemEx (copper chelate)**

Copper-deficient patients have a significantly slower granulocytopoiesis and erythropoiesis and their bone marrow biopsy specimens reveal a reduction in the number of mature granulocytes and increase in the number of promyelocytes and myelocytes compared to people without this deficiency [96, 97]. This observation led to a hypothesis that copper deficiency affects the differentiation of myeloid progenitors. Later, a StemEx component of a culture system was developed whose action is based on the effect of low copper concentrations on the differentiation of hematopoietic stem cells in vitro. In StemEx, a copper chelator, tetraethylenepentamine interacts with early and late hematopoietic cytokines [98, 99]. The use of the StemEx technology involves the expansion of cells from one unit of the UCB in the presence of StemEx for 21 days. The other portion of the UCB is left intact, and they are administered together with the cells amplified in the presence of the StemEx [100]. This approach has improved several important clinical outcomes compared to intact UCB, indicating the effectiveness of this cultivation system for the amplification of UCB HSPC ex vivo [101].

**NiCord expansion**

The NiCord technology is based on the action of an epigenetic factor, nicotinamide, which slows down the differentiation and increases the functionality of hematopoietic stem and progenitor cells obtained during *ex vivo *expansion. The addition of nicotinamide, together with hematopoietic cytokines, to the culture increases the proportion of CD34^+^CD38^-^ primitive cells and enhances migration towards SDF-1 *in vitro*. In addition, highly efficient engraftment of the amplified cells has been demonstrated in *in vivo *models [102]. NiCord not only increases the number of HSPCs compared with the technologies presented above, but also promotes efficient engraftment of cells. The particular feature of a NiCord graft is that after a 21-day expansion it contains, in addition to a HSPC fraction, a fraction of uncultivated T-cells, which is collected and re-frozen after cryopreservation. Therefore, a NiCord graft retains its immunological potential, which improves engraftment and immunological reconstitution. The results of a clinical application of HSPCs that were amplified according to the NiCord protocol and transplanted together with an additional unit of the UCB indicate an earlier recovery of neutrophils (median of 11 days vs. 25 days, *p *= 0.001) and platelets (30 days versus 41 days, *p *= 0.012) compared to the controls [103]. This study confirms the presence of long-term repopulating cells and short-term repopulating cells in the umbilical cord blood transplant after NiCord expansion.

**Expansion in the presence of Stem-Regenin 1**

Stem-Regenin 1 is a purine derivative that promotes* ex vivo *expansion of HPSCs [104]. The Stem-Regenin 1 technology uses a fractionated CD34^+^ population of UCB cells to initiate the cell culture. It has been shown that 3 weeks of expansion in a serum-free medium supplemented with Stem-Regenin 1, TPO, SCF, Flt- 3 ligand, and IL-6 results in 1118-fold amplification of CD34^+^ cells relative to the initial population. The removal of the Stem-Regenin 1 from the cultivation system leads to rapid differentiation, indicating the important role of this component in maintaining an undifferentiated state of the hematopoietic UCB progenitors. The cells obtained with Stem-Regenin 1 are capable of highly efficient engraftment after transplantation into immunocompromised mice, indicating that the presence of hematopoietic progenitors in them provides for early and sustained hematopoietic recovery. This technology has performed well in clinical trials and is actively studied today [105].

## STRATEGIES AIMED AT IMPROVING HSPC HOMING

Besides the described-above techniques there are also approaches to improving homing and engraftment of potential UCB stem cells which do not involve prior expansion. They represent an inexpensive and safe alternative to *ex vivo *expansion of HPSCs.

**Co-cultivation with E2 prostaglandin**

The study of hematopoiesis in *Danio rerio *fish revealed the involvement of dmPGE2 (16,16-dimethyl prostaglandin E2) in the homeostasis of hematopoietic stem cells [22]. It suggested that short *ex vivo *exposure of UCB cells to dmPGE2 would increase the “effective dose” of hematopoietic stem cells without significant toxicity for the patient. It has been shown that a shortterm incubation of HSPCs with dmPGE2 increases the number of these cells after transplantation and provides an advantage in serial transplantation with full multilineage bone marrow recovery in mice [106]. Promising results were obtained in the clinical use of dmPGE2, and the method continues to be actively developed [24].

**Fucosylation**

This approach aims to improve the homing of UCB stem cells in the bone marrow stroma. The technique is based on the fact that hematopoietic UCB stem cells do not migrate to the bone marrow as actively as adult bone marrow cells or mobilized peripheral blood cells. The reduced efficacy of homing in the bone marrow can be attributed partly to the lack of binding to adhesion molecules (P- and E-selectins), which are expressed on endothelial cells in bone marrow vessels [19]. Fucosylation of the selectin ligands expressed on UCB stem cells increases their affinity for P- and E-selectins of the hematopoietic microvasculature bed, which is crucial for enabling HSPC “rolling” [107]. The rather simple fucosylation procedure includes incubating the UCB cells with fucosyl transferase IV and its substrate GDP-fucose for 30 min at room temperature. The increased efficiency of UCB stem cells engraftment has been demonstrated in* in vivo *models for the use of pre-transplantation *ex vivo* fucosylation in immunodeficient mice [25, 108].

**CXCR4-SDF-1 interaction**

SDF-1 and its receptor CXCR4 also enable HSPC homing and their retention in the bone marrow. CXCR4 is expressed in different cells, including MSCs, endothelial cells, and various hematopoietic cell subpopulations, including HSCPs. SDF-1 is a potent chemoattractant for CD34^+^ HSPCs, which subsequently migrate to the bone marrow along the SDF-1 gradient after transplantation [109-113]. The optimum expression of CXCR4 in HSPCs and the effective level of SDF-1 in the recipient bone marrow support the engraftment. Dipeptidyl peptidase-4 (DPP4) is a down-regulator of this interaction, since it can cleave the N-terminal dipeptide from SDF-1, thereby reducing its activity and ability to interact with the receptor. Inhibition of this enzyme has resulted in a 2- to 3-fold increase in the homing of human CD34^+^ and Lin^-^ cells in transplantation into NOD/SCID/B2mnull mice [114]. Furthermore, it is known that dipeptidyl peptidase-4 regulates the function of hematopoietic growth factors. Therefore, inhibition of this enzyme improves not only the homing, but also cell growth mediated by growth factors [115]. The use of drugs that inhibit dipeptidyl peptidase-4 has demonstrated encouraging results for the engraftment of UCB transplants [116]. Further studies are aimed at determining the optimal dosage and timing.

**Component of C3a complement**

A C3a fragment is a product of the proteolytic cleavage of the complement protein C3. Along with numerous immunoregulatory properties, C3a sensitizes human hematopoietic stem and progenitor cells to homing towards SDF-1 via binding of C3a to the CXCR4 receptor. C3a, along with DPP4 and hyaluronic acid, fibronectin and fibrinogen, regulates the expression of SDF-1 on HSPCs [117, 118]. Preclinical studies have shown that incubation of hematopoietic stem cells with C3a prior to transplantation to lethally irradiated mice accelerates engraftment [20, 21]. However, the results of clinical application were not as successful, since C3a did not provide any advantages in terms of engraftment [23].

## CONCLUSION

Despite numerous studies aimed at optimizing the enrichment of hematopoietic transplants with stem cells, no optimized technology for the amplification of stem cells has been developed to date. The main challenges for researchers include the need for a better understanding of the composition and biological properties of the hematopoietic transplants that are responsible for hematopoietic recovery in a recipient and the development of approaches that enable the amplification of HSPCs.

A comparative analysis of data reveals two trends: the application of stromal feeder layers in systems for amplifying UCB cells or the use of various combinations of hematopoietic cytokines. However, suspension cultures in which the maintenance of hematopoietic precursors occurs only through hematopoietins do not take into account the role of the local microenvironment (interactions with stromal cells and oxygen regulation) even though it has been shown that these factors may be critical for the development of blood cells. The expansion of UCB HSPCs in co-culture is more effective than in a suspension culture. In addition, cocultivation improves the engraftment of the amplified cells after transplantation. The addition of exogenous cytokines to the co-culturing system further supports the expansion of HSPCs. Thus, it seems appropriate to use *ex vivo *systems, which include both the stromal sublayer, physiological level of oxygen, and the necessary cocktail of cytokines and growth factors, for amplification.

**Fig. 4:**
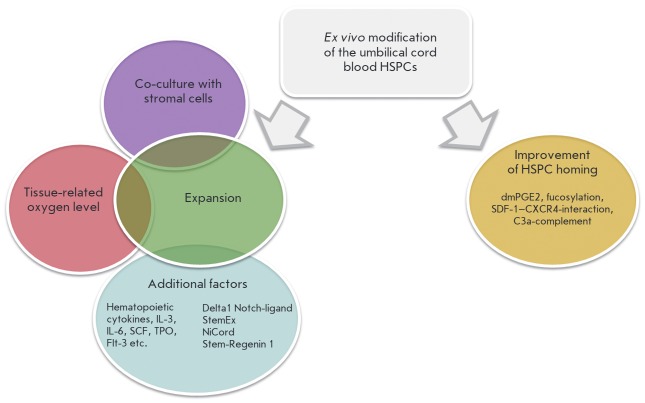
Current technological approaches to the modification of hematopoietic stem and progenitor cells from umbilical cord blood *ex vivo*

Molecular genetic approaches have proven to be quite successful, as well; they are aimed at both amplification of hematopoietic cells and improvement of the homing of transplanted cells in a recipient’s bone marrow (*Fig. 4*). *Ex vivo *systems for the amplification of HSPCs have already been developed and successfully used: however, the search for new effective approaches to UCB cells expansion that are based on modern cellular and molecular biological techniques continues.

## Abbreviations

UCB: umbilical cord blood
HSPC: hematopoietic stem and progenitor cells
MSC: mesenchymal stromal cells
CFU: colony-forming units
G-CSF: granulocyte-colony stimulating factor
GM-CSF: granulocytemacrophage colony-stimulating factor
IL-2: interleukin 2
IL-3: interleukin 3
IL-4: interleukin 4
TGF-β: transforming growth factor beta
dmPGE2, 16,16: dimethyl prostaglandin E2
SDF-1: stromal cell-derived factor-1
CXCR4: CXC chemokine receptor type 4
C3a: complement fragment 3a
